# DIETARY IMPACT ON GUT MICROBIOTA, COGNITIVE PERFORMANCE, AND HIPPOCAMPAL MARKERS IN AGING RATS

**DOI:** 10.1093/geroni/igad104.3276

**Published:** 2023-12-21

**Authors:** Rebecca Solch-Ottaiano, Colin Harper, Elizabeth Engler-Chiurazzi, Saifudeen Ismael, Madison Prats, Milla Volic, Gregory Bix, Demetrius Maraganore

**Affiliations:** Tulane University School of Medicine, New Orleans, Louisiana, United States; Tulane University School of Medicine, New Orleans, Louisiana, United States; Tulane University School of Medicine, New Orleans, Louisiana, United States; Tulane University School of Medicine, New Orleans, Louisiana, United States; Tulane University School of Medicine, New Orleans, Louisiana, United States; Tulane University School of Medicine, New Orleans, Louisiana, United States; Tulane University School of Medicine, New Orleans, Louisiana, United States; Tulane University School of Medicine, New Orleans, Louisiana, United States

## Abstract

Many patients with mild cognitive impairment have alterations in gut microbiota composition and dietary consumption directly modulates gut microbiota; therefore, the quality of a diet may impact cognitive function. Twelve-month-old male Fischer 344 rats (n=10/group) were assigned to a Mediterranean diet (MeDi), Western diet (WD), or Chow for three months to determine the effect of diet in aged animals. Animals underwent neurobehavioral assessments including the Morris water maze (MWM), and radial arm water maze (RAWM). Fecal samples were analyzed via 16S rRNA sequencing. Markers of barrier and immune function in the hippocampus were measured via qPCR. The MeDi (p=0.06) and Chow (p=0.003) groups had a trend to or swam a shorter distance compared to the WD in the MWM. There was no difference in RAWM total errors between Diets (p=0.43). Microbiota analyses exhibited differences in alpha- and beta-diversity. Chow diet had a higher Simpson and Shannon index compared to MeDi (p< 0.02) and WD (p< 0.001). The MeDi (p=0.03) and Chow (p< 0.001) groups had a higher Chao1 index compared to the WD group. Beta-diversity differed by Diet for weighted UniFrac (p< 0.03), and unweighted UniFrac (p< 0.001). For barrier integrity, there was a trend for Occludin expression to differ by Diet (p=0.07) but Claudin-5 did not differ (p=0.81). For immune function, the WD group had a trend to have a higher expression of GFAP compared to the MeDi (p=0.05) but a lower Iba-1 expression (p=0.009). A short-term diet had a subtle effect on cognitive performance but modulated microbiota compositions and hippocampal immune marker expression.

